# Expand Combined Internal Medicine-Pediatrics Fellowships to Address Pediatric Subspecialty Shortage

**DOI:** 10.7759/cureus.116276

**Published:** 2026-09-15

**Authors:** Michael J Aronica, John Szymusiak, Benjamin Doolittle

**Affiliations:** 1 Internal Medicine-Pediatrics, State University of New York, Buffalo, USA; 2 Pediatrics, University of Pittsburgh School of Medicine, Pittsburgh, USA; 3 Medicine, University of Pittsburgh School of Medicine, Pittsburgh, USA; 4 Internal Medicine-Pediatrics, Yale School of Medicine, New Haven, USA

**Keywords:** graduate medical education, internal medicine-pediatrics, pediatric fellowships, salary discrepancies, specialty shortages

## Abstract

Currently, the pediatric subspecialties are facing a workforce crisis that threatens access to specialized care for children with complex and chronic medical conditions here in the United States. Many pediatric subspecialty fellowship programs struggle to fill training positions, raising concern about the sustainability of the future workforce in those subspecialties. The factors contributing to the shortage have been posited as relating to lower compensation and higher rates of burnout. Compared to Internal Medicine subspecialists, many pediatric subspecialties offer significantly lower compensation and longer training. We propose that increasing the number of opportunities for graduates of Internal Medicine Pediatric residencies to enter combined Internal Medicine-Pediatric Fellowships will help offset the decreasing number of pediatric subspecialists.

.

## Editorial

The problem: pediatric subspecialist shortage

There is a crisis among the pediatric subspecialists: there are not enough of them. In many specialties, there are not even enough applicants to fill available fellowship training spots, which places further strain on the future workforce [1]. There are significant clinical shortfalls of pediatric subspecialists in endocrinology, nephrology, and rheumatology [2]. There is also significant variation across the United States: eleven subspecialties have one or fewer physicians per 100,000 children in some referral regions [3]. Subspecialists tend to concentrate in urban areas, leaving those in rural or even suburban areas without access [3]. According to a study by the Children’s Hospital Association, children must wait more than 13 weeks for specialist consultation for genetics, developmental-behavioral pediatrics, neurology, and dermatology [4]. This represents a crisis for our children's care - especially for those with complex, chronic illness [5].

Reasons for the pediatric subspecialist shortage

When it comes to medical specialties, subspecialist supply is a uniquely pediatric problem. This is clearly demonstrated when the internal medicine (IM) and pediatrics (Peds) fellowship match data are compared. In 2025, the IM fellowships of endocrinology, gastroenterology, and infectious disease and filled at a rate of 98%, 99%, and 70% respectively. Comparably pediatric endocrinology, gastroenterology, infectious disease and filled at rates of 60%, 81%, and 48% respectively [6].

One important pressure behind this shortage is well-known: money [4-5,7,8]. For example, according to benchmarks from the Association of American Medical Colleges (AAMC), seven pediatric specialties at the assistant professor level make less than their general pediatrics counterparts: adolescent medicine ($12,000 less), developmental pediatrics ($4,000 less), endocrinology ($9,000 less), genetics ($4,000 less), infectious disease ($19,000 less), nephrology ($6,000 less), and rheumatology ($11,000 less) [9]. Catenaccio et al. showed that a developmental pediatrician will earn $1.9 million less than a general private practice pediatrician and $1.1 million less than a general academic pediatrician [10]. Prolonging training to earn less money than a general pediatrician is likely a deterrent to prospective applicants who are on the fence about a pediatric subspecialist career. 

One solution: the med-peds combined specialist

There is a partial solution: the internal medicine-pediatrics (med-peds) physician. Med-peds residents complete four years of training and are eligible to sit for both the pediatric and IM boards. They also are eligible for both pediatric and IM subspecialty training [11]. Career data show that 34.7% of med-peds physicians choose subspecialty training, but only 9.2% pursue exclusively pediatric subspecialty training [12]. Most med-peds physicians care for both children and adults, either as an ambulatory provider (16.2%), med-peds hospitalist (14.6%), academic (10.2%), or as a combined subspecialist (8.54%) [12]. Given the broad commitment of med-peds physicians to care for both adults and children, increased opportunities to pursue dual fellowship training shows great promise to expand the pediatrics subspecialty workforce.

The med-peds physician has one of the most versatile trainings. As such they can quickly adapt and provide care in various clinical settings and across the age spectrum, which makes them especially attractive in underserved areas which may not have the clinical volume to support dedicated pediatric subspecialists. Combined physicians can also use the higher reimbursement from adult subspecialty care to help offset the lower rate of pediatric reimbursement [9]. In our institutions, med-peds trained subspecialists have salaries that represent a blended rate between IM and pediatrics that is significantly higher than the pediatric subspecialty rate. We believe that increasing combined subspecialty training programs would increase the pipeline and alleviate the shortage of pediatric subspecialists. Especially in the most under-represented subspecialties, even a few more providers could make a substantial difference. While we accept that some specialties facing workforce shortages such as child development and child abuse do not have analogs on the adult side, many of the at-risk subspecialities such as rheumatology and nephrology do.

Existing barriers to med-peds combined specialists

We believe that many med-peds trainees would welcome dual-training if existing barriers could be removed by Graduate Medical education (GME). Dual fellowship training offers much promise with little downside. Most of the barriers to expanding the combined fellowship workforce are logistical. The American Board of Internal Medicine (ABIM) and the American Board of Pediatrics (ABP) recommend that combined programs subtract one year from the combined duration of the two individual training pathways to account for overlap in the scholarly components of the categorical fellowships [6]. This means that a med-peds physician pursuing an adult fellowship would only need to increase their training by one year to be eligible for the pediatric boards. This helps alleviate funding issues: a med-peds fellow is cheaper to train than two separate medicine and pediatric fellows since the duration of training is shorter and onboarding costs are reduced. Despite these guidelines, the boards do not approve combined training programs; they instead consider proposals on an individual basis. This adds a significant barrier for programs that do not have existing pathways in place for combined fellows. Moreover, this places a significant burden on the applicant and the fellowship directors to ensure that proposed training pathways meet requirements.

The match process also introduces significant barriers for potential applicants. While the adult and pediatric fellowship matches occur over similar periods, an applicant cannot enroll in both the adult match and pediatric match [6]. This causes challenges when applicants interview for several potential combined positions. If some positions utilize the adult match and others the pediatric, then the applicant is unable to rank all potential programs. Thankfully, these barriers could be easily remedied if the ABIM and ABP provided more standardized combined training pathways. For example, the combined fellowships would agree to use either the adult or pediatric match exclusively.

Next steps

The shortage of pediatric subspecialists requires an innovative, sustainable solution. Reducing logistical barriers to med-peds fellowship training affords an opportunity to increase the pediatric subspecialty workforce in these critical areas with very little cost and without major changes to the existing system. Additional incentives, such as stipends to supplement the extra years of dual fellowship training or clinical pathways with fewer research requirements could further attract med-peds trainees. Broader policy reform to improve pediatric healthcare compensation and move towards pay equity with adult subspecialists is also critical. Consideration should also be made to offering a program similar to the National Health Service Corps to attract combined subspecialists to rural areas and reduce inequities in access.

Expanding dual training programs might not solve the problem, but it offers a promising pathway to increase subspecialty pediatricians. Med-peds fellowship pathways can be a cornerstone of a broader strategy to ensure every child has timely access to expert care. Expanding and supporting these pathways offers a meaningful strategy to strengthen the pediatric subspecialty workforce and improve expert care for all children.
